# Case Report: Lessons learned from large animal implantation of an all-natural tissue engineered vascular graft

**DOI:** 10.3389/frtra.2025.1676566

**Published:** 2025-10-07

**Authors:** Alexandru I. Dumitru, Bryan T. Wonski, Renée A. Cole, Mitchell R. Weaver, Kelsey C. Carpenter, Loay S. Kabbani, Mai T. Lam

**Affiliations:** ^1^ Department of Biomedical Engineering, Wayne State University, Detroit, MI, United States; ^2^ Division of Vascular Surgery, Department of Surgery, Henry Ford Hospital, Detroit, MI, United States; ^3^ Division of Laboratory Animal Resources, Wayne State University, Detroit, MI, United States

**Keywords:** vascular graft, tissue engineering, large animal model, abdominal aorta, surgery, tissue engineered vascular graft, rabbit model

## Abstract

Cardiovascular disease continues to be the number one cause of morbidity and mortality across the world. Coronary artery bypass graft (CABG) procedures are the most commonly performed major surgery in the U.S. Grafts are difficult to source as patients do not have many sites from which to harvest donor tissues as autografts. Plastic grafts have issues of infection and are only used as a last resort. Tissue engineered vascular grafts have potential to solve the need for all-natural vascular grafts in the clinic. In this study, we evaluate the feasibility of a completely biological engineered vascular graft for implantation in a large animal model of a rabbit. An all-biological tissue engineered graft was grown in our laboratory, composed of a tunica adventitia derived from human dermal fibroblasts and a tunica media made from human aortic smooth muscle cells. The all-biological engineered graft exhibited the “look and feel” of a natural vessel. The engineered graft was implanted into the abdominal aorta of a New Zealand rabbit. The graft easily anastomosed to the native abdominal aorta and showed no leakages. Once reperfused, the graft was able to withstand blood flow briefly, prior to exhibiting dissection between the media and adventitia. Color doppler ultrasound showed flow through the abdominal aorta, however, not through the graft region due to the dissected layers creating a blockage. These results support a shift from the traditional paradigm of designing vascular grafts to mimic the multi-layered native structure. The two-layer engineered graft tested here exhibited dissection between the layers, a phenomenon that has yet to be reported in the field to our knowledge. Based on these findings, we recommend a single layer engineered graft to best prevent dissection.

## Introduction

Cardiac disease continues to dominate the medical field in cases of morbidity and mortality. Each year, approximately 400,000 coronary artery bypass graft (CABG) surgeries are performed in the United States, making it the most common major surgical procedure (1). During these procedures, veins are most often harvested from the patient as autograft material, since arteries are in extremely limited supply. Of vein procedures, 40% fail within 10 years as they are not built to withstand arterial pressures and biomechanics. To solve this issue, tissue engineering of arteries has great potential to serve this significant need.

In this present study, an all-biological engineered arterial graft was explored as a viable option for arterial repair. Our tissue engineered blood vessel was created using our laboratory's unique method for constructing blood vessels *in vitro*. Our methods incorporate cell sheets formed into ring structures that are stacked to form a tubular vessel (2–4). The engineered vessels are constructed completely of cells and naturally degrading hydrogel, making them all-biological. The vessels used in this study were composed of the tunica adventitia and tunica media layers, using fibroblasts and smooth muscle cells, respectively, to serve as the primary vasculature. These two arterial layers were chosen in order to provide structural integrity and strength to withstand suturing and blood pressures. The engineered artery was tested for anastomosis, suturability and hemodynamics in a large animal rabbit model. A surprising finding in this work was that once subjected to blood flow, the bi-layered engineered vessel delaminated between the two layers, causing the vessel walls to create a flap which subsequently obstructed flow. This study shows that a two-layered tissue engineered vessel, although physiologically-accurate, may not be the optimal construct to serve as a vascular graft. From here, our team will explore single-layer engineered vessels as a better option. Our newly founded information is vital to the field to push towards a viable vascular graft solution for patients.

## Methods

### Tissue engineered vessel protocol

The tissue engineered arteries were made using a previously established protocol by our laboratory, termed all-biological engineered blood vessels (BEBVs) (2–4). Briefly, vascular cells are seeded in a plate around a central 3D printed plastic post. The cells form a cell sheet which is engineered to detach from the bottom of the plate from a layer of hydrophobic poly(methylsiloxane) (PDMS). The cell sheet aggregates around the post, creating a tissue ring. The vascular cell rings are subsequently stacked to form the final tubular vessel. The engineered vessel prepared for implantation was a bilayer vessel composed of a tunica media and adventitia. Our methods can create vessels of any desired size, i.e., diameter and length (5). The lumen of the engineered vessel created was 3 mm in diameter, to match the rabbit abdominal aorta diameter of 3–4 mm as closely as possible. The length of the vessel was approximately 2.2 cm long and composed of 66 rings of engineered vascular tissue.

**Figure 1:**
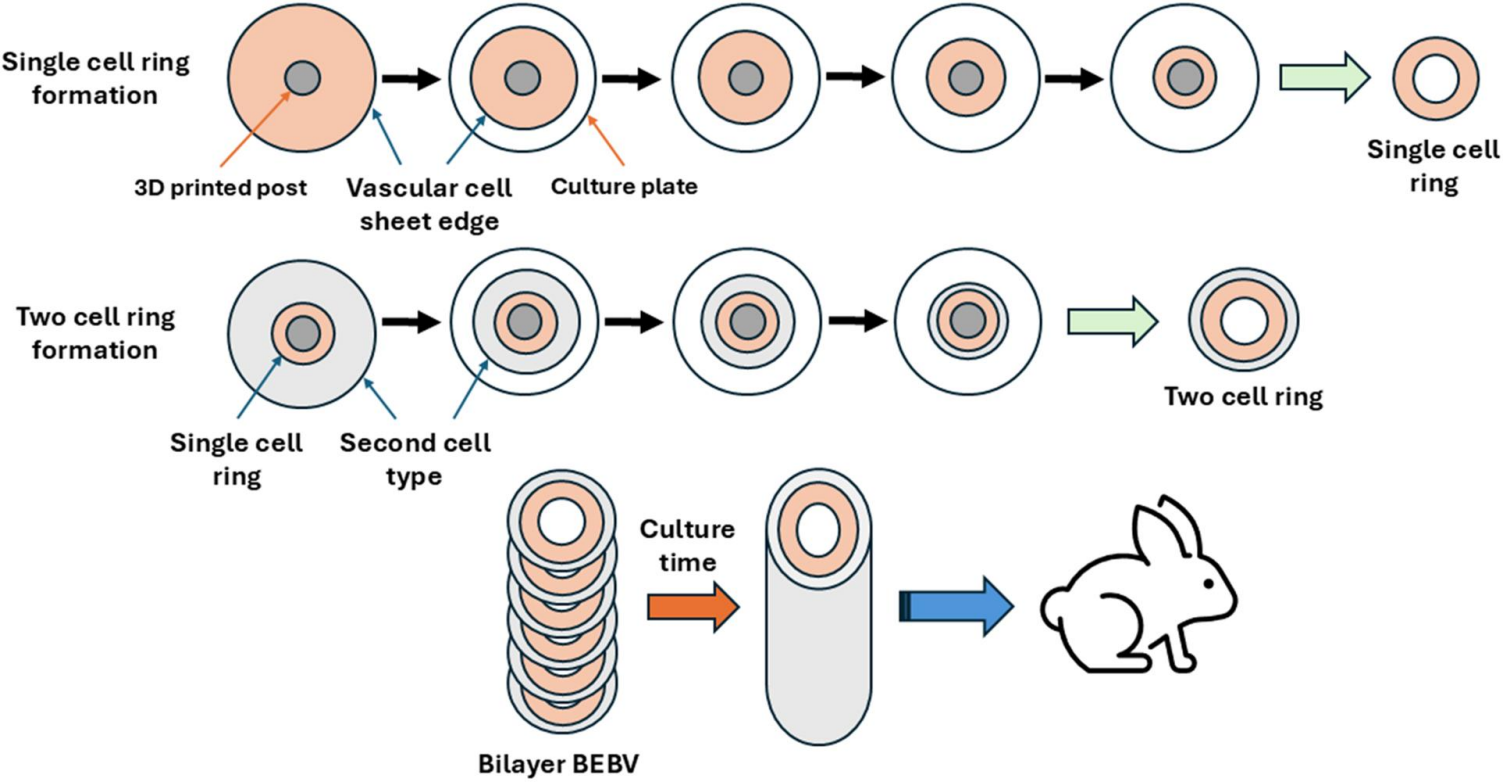
Diagram of construction of the biological engineered blood vessel (BEBV) with a bilayer. Vascular rings were formed in a traditional culture plate by seeding vascular cells into the plate around a central 3D printed post with a diameter determining the final vessel lumen size. The resultant cell sheet detaches from the bottom of the plate, and subsequently aggregates toward the center of the plate around the post to form a tissue ring. First, a single cell ring is formed, followed by formation of a second cell ring around the first ring in the plate, creating two-cell rings (i.e., bilayer rings). The bilayer rings were stacked into a tubular vessel and allowed to culture for 14 months to allow ring-to-ring growth and integration. The BEBV was then implanted into a large animal rabbit model.

**Figure 2:**
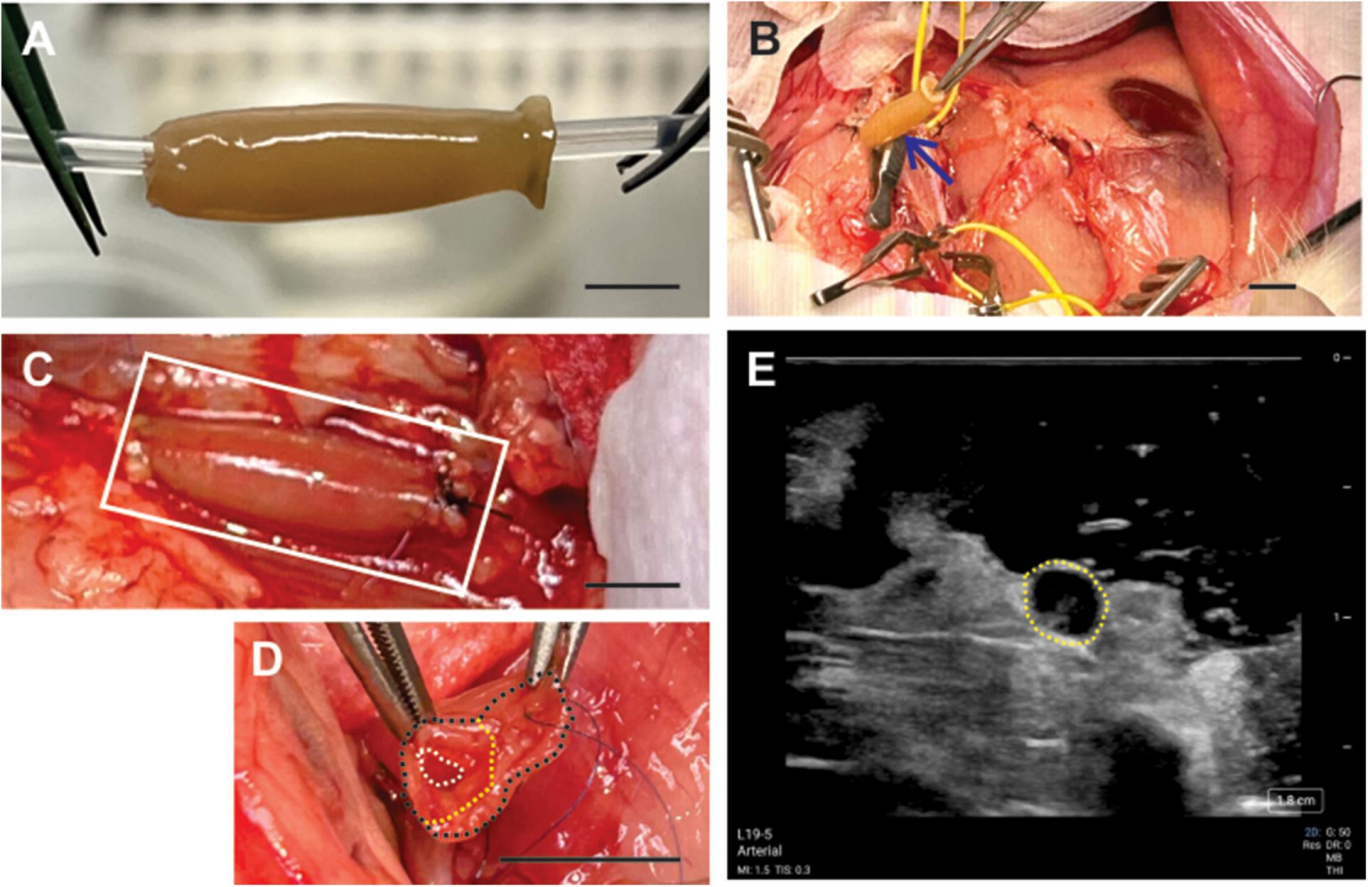
All-natural tissue engineered vascular graft implanted into a rabbit abdominal aorta model. **(A)** Engineered graft built in the laboratory composed of human cells; total length was 2.2 cm. **(B)** Surgical site of the graft (blue arrow). **(C)** Anastomosed graft (white box) to the abdominal aorta. **(D)** Graft dissected between its two layers following blood perfusion, marked by the outer vessel wall (black dashed line), inner lumen (white dashed line), and the dissected region (yellow dashed line). **(E)** Ultrasound showed vessel dissection within the vessel lumen (yellow dashed line). Scale bars = 1 cm.

In order to create the bilayer structure, first, vascular rings of human smooth muscle cells (SMCs) were created, followed by formation of human fibroblast rings around the SMC rings *in situ* (Figure 1). The bilayers rings were stacked into a vessel and allowed to culture to allow for ring-to-ring growth, integration and extracellular matrix deposition. The final bilayer vessel was cultured for 14 months then implanted into a rabbit model (Figure 2).

### Human cell sources

The tunica adventitia was composed of human dermal fibroblasts extracted from human skin donated from a local plastic surgery clinic. All patient tissues were obtained with consent in accordance with the guidelines set by the Institutional Review Boards of Wayne State University and the Henry Ford Hospital System (IRB # 054514M1E). The tunica media was composed of human aortic smooth muscle cells (HASMCs) purchased commercially (PCS-100-012, ATCC, Manassas, VA). The intima was not included in the vessel tested here as the grafts were designed to rely on endogenous endothelization in the future after implantation into patients. Here, an implantation time of 1 h was planned in order to investigate anastomosis and hemodynamics of the engineered vessel. Immunogenicity testing would entail longer implantation times and is not a focus of this study, thus the use of human cells in the rabbit model was not an issue as signs of immune rejection would not be observed in the study time period of 1 h.

### Surgical technique

The engineered graft was anastomosed to the abdominal aorta (AA) of a female New Zealand white rabbit about 3.45 kg in weight and of good health. All procedures were performed in accordance with the Institutional Animal Care and Use Committee of Wayne State University (protocol #23-08-6043). Implantation time was set to 1 h in a non-survival procedure. Briefly, animals were given buprenorphine (0.01–0.05 mg/kg) subcutaneously, followed by sedation with ketamine (10–40 mg/kg intramuscularly) and xylazine (1–5 mg/kg intramuscularly) and then anesthetized with isoflurane for the duration of the procedure. The abdomen was shaved and disinfected with betadine for surgical prep. Lidocaine (2 mg/kg) and bupivacaine (1 mg/kg) were injected equidistantly approximately 0.5 cm apart in an ellipse around the incision sites prior to making the surgical incision. Vascular access was obtained by a vertical abdominal incision through the skin and linea alba at the midline. Saline-moistened vessel loops were looped around the artery at proximal and distal regions of the aorta cutdown. Vascular clamps were applied proximally and distally.

The abdominal aorta was controlled proximally and distally with atraumatic vascular clamps. A segment of the aorta between the clamps approximately 2.4 cm in length was excised. The resected portion of the aorta was replaced with an engineered graft, first performing the proximal anastomosis with running 7–0 prolene suture in an end-to-end fashion and then performing the distal anastomosis with running 7–0 prolene suture. Immediately prior to completion of the distal anastomosis, the graft and artery were vented to remove residual air. End-to-end anastomosis was performed with consistent assurance of intimal eversion of the native artery during suturing. The distal anastomosis was completed and the vascular clamps removed to restore flow.

Color doppler ultrasound was taken of the implanted vessel. The probe was gently placed on top the engineered vessel directly for 30 s at a time to minimize vascular damage. The abdomen was periodically wetted with saline during the open imaging procedure. Once imaging was completed, the animal was immediately humanely euthanized with an overdose of isoflurane and thoracotomy.

## Results

The all-biological engineered graft exhibited the “look and feel” of a natural vessel (Figure 2A). The graft demonstrated suture retention strength as shown by successful anastomosis to the AA without signs of leakage (Figures 2B,C). The graft exhibited compliance similar to the native AA to create a seamless transition once anastomosed. Blood loss was minimal once un-clamped with no leaking from the sutured ends nor along the length of the vessel. For a brief period, the vessel pulsated under blood flow (Figure 2C) until dissection between the adventitia and media layers of the graft was observed (Figure 2D). Under color doppler ultrasound, no thrombotic occlusion was observed, although the dissection between the two layers was seen at the distal end (Figure 2E) which blocked flow through the graft.

## Discussion

Overall, the all-natural engineered graft construction proved viable as a vascular substitute and was able to achieve timely hemostasis once implanted into a large animal model. The traditional paradigm that vascular grafts should replicate the native multilayer structure (1, 6) is challenged by the results of this study. Here, we find that a multilayer graft resulted in issues with dissection, a phenomenon that has yet to be reported in the field. Hence, this study supports a paradigm shift towards single-layer grafts. This study provides pertinent information for vascular graft development to optimize translatability.

A rabbit model was chosen as the first level of large animal model (7) with the subsequent goal of progressing up animal models (e.g., pig). Rabbit models have been used in cardiac research for some time, due to the larger size of arteries available for implantation compared to commonly used rodent models (7, 8). Rabbit myocardium is more similar to humans compared to rodents (9).

The structure of the engineered vessel chosen for this study was based on replicating the main structural components of a blood vessel- the tunica adventitia and media. The mechanical aspects of the vascular graft were focused on to address immediate issues of providing a structural conduit for blood flow. A version of our vessels does implement the intima layer. However, in the current acute study with a 1 h implantation, the mechanics of the vessel and anastomosis were the main testing parameters. Hence, the adventitia and media layers constituting the primary strength components were focused on without interference from the added variable of the intima. Previously, we have reported mechanical data on multiple versions of our engineered vessels, demonstrating necessary strength requirements to serve as an arterial graft. We have previously reported a maximum tensile strength of 1,500 ± 334 kPa and maximum burst pressure of 229 ± 23.8 mmHg (10).

Longer culture periods promote extracellular matrix deposition, a method often used to strengthen engineered tissues (11, 12). Hence, we tested a long 14 month culture period to create a stronger tissue. The primary purpose of a longer culture period was to promote ring-to-ring integration via extracellular matrix deposition and cell proliferation within the layers. Cross layer integration is not desired as that scenario constitutes a disease state, that is of cells intermixing across layers. Interlayer cell mixing has not been observed in our bilayer engineered vessels (3, 4). The mechanics of the vessel were not fully tested due to the almost immediate layer dissection, showing that in this situation graft structure outweighed mechanical factors.

Pre-endothelialization of the graft was not an aspect of this study as the focus was to test the form that would be used directly as a patient graft in the future. In order to create a graft compatible to human implantation, our lab has been able to source human fibroblasts and human cardiac smooth muscle cells for creating the adventitia and media, respectively. There does not currently exist an autologous human source of arterial endothelial cells available for a graft. Advantageously, the body naturally endothelializes implanted conduits, providing an endogenous intima within about 4 weeks as shown in *in vivo* studies (13, 14). This natural phenomenon provides endogenous, autologous endothelialization needed for a graft, hence minimizing the need for pre-endothelialization.

Based on this study's results, the bilayered engineered vessel showed issues of delamination between the layers once blood flow was reestablished. The evident acute failure of the graft was due to the boundary between the adventitia and media layers separating under flow pressure. The graft's failure occurred well before the mechanics of the vessel could be tested. Hence, ultimately the failure can be attributed to the layer delamination. Additionally, surgical technique was not the source of failure since delamination occurred distal to the locations of anastomosis and in a region untouched by the surgeons. These results support a single layer graft as a more effective conduit. In the literature, several multi-layered engineered vascular grafts have been created. One study did not witness delamination between layers in an *in vitro* setting (15). This stresses the importance of reporting our delamination findings following animal implantation.

Typically, in an animal implantation study, immunogenicity is a consideration when implanting a human construct into an animal for concerns of tissue rejection. Immunogenicity issues arise after sufficient implantation times. In this study, the short implantation time and non-survival surgery negated possible study of and issues of immunogenicity.

Post-implantation retrieval and analysis of the BEBV was not possible due to delamination and subsequent tissue breakdown under pressure. With future implantations, vessels will be retrieved after the study conclusion and histological and genetic analysis performed.

In conclusion, the main message of our study is that a multi-layered vascular graft has the probability of exhibiting issues of tissue delamination and vessel occlusion from tissue breakdown, which has not yet been reported to our knowledge. Single layered vascular grafts would circumvent this issue and is the basis of our current work.

## Data Availability

The datasets presented in this article are not readily available because of pending intellectual property. Requests to access the datasets should be directed to mtlam@wayne.edu.

## Supplementary material

Supplementary Video S1 Engineered graft implantation by the vascular surgeons through anastomosis to the rabbit abdominal aorta.
